# Defining Barriers and Facilitators to Advancement for Women in Academic Surgery

**DOI:** 10.1001/jamanetworkopen.2019.10228

**Published:** 2019-08-30

**Authors:** Julie A. Thompson-Burdine, Dana A. Telem, Jennifer F. Waljee, Erika A. Newman, Dawn M. Coleman, Hadley I. Stoll, Gurjit Sandhu

**Affiliations:** 1 Department of Surgery, Michigan Medicine, Ann Arbor

## Abstract

**Importance:**

Sex equity is elusive in academic surgery departments across the United States. Persistent inequities remain a considerable problem and inhibit professional advancement for female surgeons. Identifying the factors that promulgate sex discrepancies may provide a framework for institutional growth and personal progress for women.

**Objective:**

To identify barriers and facilitators to success at the individual and organizational level to develop evidence-based interventions designed to close the sex gap in surgery.

**Design, Setting, and Participants:**

This qualitative study included 26 female participants who were current and former surgical faculty employed by Michigan Medicine, the health system of the University of Michigan, between 2000 and 2017. Semistructured personal interviews were conducted from June 28 to September 29, 2017, via telephone. Each interview lasted 45 minutes to 1 hour. Interviews were recorded and then transcribed for analysis.

**Main Outcomes and Measures:**

The interview included 7 questions referring to the surgeon’s experience with the Michigan Medicine Department of Surgery and 7 questions referring to nonspecific areas of interest.

**Results:**

The 26 participants in this study ranged in age from 32 to 64 years, with faculty experience ranging from 3 to 22 years. Thematic analysis was used to locate, analyze, and report patterns within the data related to barriers and facilitators for women in academic medicine. Three major themes were identified by researchers. Participants reported that (1) organizational culture and institutional policies affect opportunities for advancement; (2) relational interactions with leadership, mentors, colleagues, and staff affect promotion and attrition; and (3) individual characteristics mediate the perception of professional and personal success.

**Conclusions and Relevance:**

In this qualitative study of 26 female academic surgeons, a complex matrix of organizational and individual factors were found to contribute to sex inequities in academic surgery. This research may provide insight into the sex biases that inhibit advancement, may inform strategies that facilitate progress, and may inspire interventions that could help eliminate institutional and individual barriers to the academic success of women.

## Key Points

**Question:**

What are the perceived barriers and facilitators to promotion and professional success for women in academic surgery?

**Findings:**

In this qualitative study of 26 female academic surgeons, a complex matrix of organizational and individual factors was found to contribute to sex inequities for the professional advancement of women in academic surgery.

**Meaning:**

This research may provide insight into the sex biases that inhibit advancement, may inform strategies that can facilitate progress, and may inspire interventions that could help eliminate institutional and individual barriers to the academic success of women.

## Teaser

This qualitative study describes female surgeons’ perceptions of factors promoting and hindering their professional success in an academic setting.

## Introduction

Advancing sex equity is an urgent need for US surgery departments. Exclusion of women in academic surgery equates to losses of health care workforce resources, team effectiveness, and cognitive diversity for institutions.^1,2^ Fundamentally, the persistence of sex inequity is associated with hindrance of female surgeons in their research and clinical practice, decreased professional advancement, career stagnation, and ultimately attrition from surgical careers.^3^

Women represent only 19% of tenured professors, 12% of department chairs, and 11% of medical school deans in the United States.^4,5,6,7^ A survival analysis of all Michigan Medicine faculty members from 2005 to 2015 revealed that women were significantly less likely to advance to associate professor from assistant professor. In a survey of women across all departments from our institution, female faculty reported lower professional satisfaction compared with men, were less likely to hold tenure-track or fully tenured positions, and were more likely to choose a non–tenure track position owing to concerns regarding work-life balance.^8^ The Michigan Medicine Department of Surgery is actively implementing strategies to shift negative sex paradigms of years past to purposefully foster the advancement of female surgeons. In 2017, the Michigan Promise was launched as a comprehensive 6-part faculty and resident development initiative with a central tenant to build a diverse and inclusive culture where individuals cannot only advance, but also thrive. The Michigan Promise is an initiative coordinated through the newly created Office of Faculty and Resident Life that pledges to support each individual to achieve their full professional and academic potential. The Michigan Women’s Surgical Collaborative (MWSC) was established to specifically support and develop female academic surgeons. The MWSC is now affiliated with the Michigan Promise in a partnership to facilitate sex equity.

Documenting female surgeons’ experiences prior to this transition and as it unfolds will be critical for understanding which pathways are most beneficial for advancement. In a surgical culture where women disproportionally struggle to achieve success without expressly making the feminine visible in a traditionally masculine setting, we endeavored to collect personal stories that acknowledge the lived experiences of female surgeons.^9,10^ Through these women’s narratives, we sought to pinpoint challenges that constrain their careers and uncover avenues of promotion and growth. By identifying these barriers to success and facilitators to progress, we hope to aid in the development and refinement of evidence-based interventions designed to close the sex gap in surgery.

## Methods

### Study Design

This qualitative case study was designed to identify barriers and facilitators to advancement of female academic surgeons in the Michigan Medicine Department of Surgery.^3^ A case study is an empirical investigation of a phenomenon in depth and within real-life context.^11^ We use this approach to query the experiences shared by participants. The study was approved by the Michigan Medicine Medical Institutional Review Board. Each participant was informed about the scope of the study and consented for participation. This study followed the Standards for Reporting Qualitative Research (SRQR) reporting guideline.

Because of the sensitive nature of the information, additional precautionary measures were taken to ensure external and internal confidentiality for participants. The integrity of sensitive information was retained and presented using appropriate fictionalizing techniques of nonfiction data.^12^ Researchers verified data representations reported in study publications by member-checking with participants. Member-checking is a process in which discrete data are reviewed and approved for publication by the participant.^13^

### Setting

This study was performed at Michigan Medicine, the academic medical center of the University of Michigan. The Department of Surgery includes more than 130 faculty across more than 20 specialties. Currently, 22% of the surgical faculty are women. There are 8 section heads at Michigan Medicine, all of whom are men.

### Participants

The participant pool included 65 women; 28 were currently employed surgical faculty with the Michigan Medicine Department of Surgery and 37 had previous surgical faculty appointments between 2000 and 2017. This participant pool was intentionally chosen because of their sex, academic appointments, and rank with the express goal of recording experiences and perspective of female surgeons to identify issues and concerns specific to this group. Potential participants were contacted via email, with a follow-up telephone call requesting participation. The overall response rate was 46%. However, owing to the sensitive nature of the information being collected, some respondents expressed concerns regarding disclosure and chose not to complete the interview process. In total, 40% of participating women completed the interview. Among participants, the duration of employment in the University of Michigan Department of Surgery ranged from 4 to 7 years. Expressed reasons for attrition varied, including perceived lack of opportunity, hostile work environment, better opportunities elsewhere, job opportunities for partners outside of Michigan, and health compromised by stress.

### Measures

Informed by an extensive literature review looking at sex disparities for female faculty in surgery, the content of the interview was developed jointly by a leadership coach and members of the MWSC. The interview included 7 items referring to the surgeon’s experience with the Michigan Medicine Department of Surgery along with 7 items about nonspecific areas of interest. Each interview lasted 45 minutes to 1 hour. Interviews were recorded then transcribed for analysis.

### Data Collection

From June 28 to September 29, 2017, semistructured personal interviews were conducted via telephone. The leadership coach conducted the interviews with participants to ensure anonymity and protect their confidentiality. The interview format was closely followed, with a generous allowance for personal stories to be shared. The interviewer collected information that included recruitment to Michigan Medicine, starting faculty position, start-up package, salary offer, faculty track, and access to resources. A promotion timeline was established, including promotion expectations, processes, barriers and facilitators, and stewards of the process. If the participant was no longer affiliated with Michigan Medicine, the interviewer inquired about factors that led to the transition. Current faculty members were asked about the breadth of their work, advancement possibilities, and leadership opportunities. Additional interview topics included mentorship and sponsorship relationships, professional visibility, personal circumstances, and perception of and career-related experiences in association with their sex.

### Data Analysis

Each interview was transcribed. All 26 transcripts were then coded using NVivo 11 software.^14^ Coding was completed by 2 surgical education researchers. These 2 researchers together developed a set of codes to capture how participants articulated their experiences. Following the initial coding process, they met to reconcile discrepancies in coding. Mindful of methodological error, principles of reflexivity were used to help guard against personal perceptions influencing their choice of coding method.^15^ The final codes were grouped into themes. The coders felt they reached informational redundancy within the sample that was collected and that the sample size was adequate for this qualitative case study.

Using an interpretivist approach, thematic analysis was conducted.^16^ The interpretivist approach provided a lens to locate, analyze, and report patterns within the data set where values related to barriers and facilitators in academic advancement for women.^16^ All qualitative analysis was conducted using NVivo Pro 11 software.^14^

## Results

Thematic analysis of the narratives of the 26 women in this study revealed archetypes of participant experiences related to barriers and facilitators associated with professional and personal advancement, as well as shared models of how participants evaluated their time as female surgeons in the Michigan Medicine Department of Surgery. The participants in this study ranged in age from 32 to 64 years and reported years of faculty experience ranging from 3 to 22 years. Participant experiences reflected change over time. The themes remained consistent for individuals across the historical timeline of this study. The perception of dynamics and sex inequities was a core feature of the investigation.

We derived a thematic structure that consisted of 3 major themes:

1. Organizational culture and institutional policies affect opportunities for advancement.
2. Relational interactions with leadership, mentors, colleagues, and staff affect promotion and attrition.
3. Individual characteristics mediate the perception of professional and personal success.

**Figure 1.**
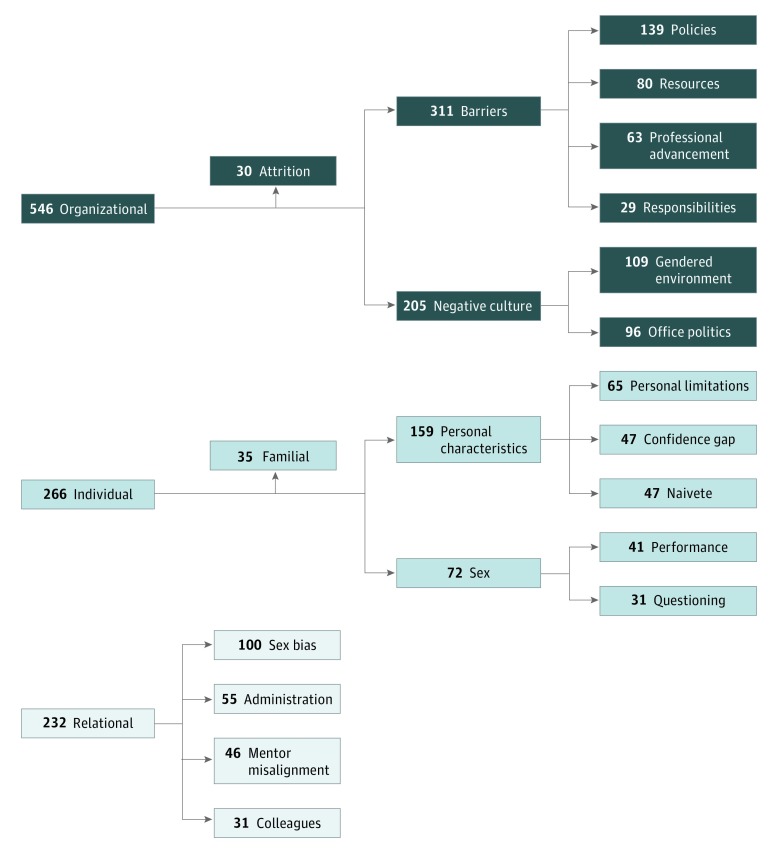
Diagram of Barrier Codes (1044 Citations): Thematic Narrative Analysis

**Figure 2.**
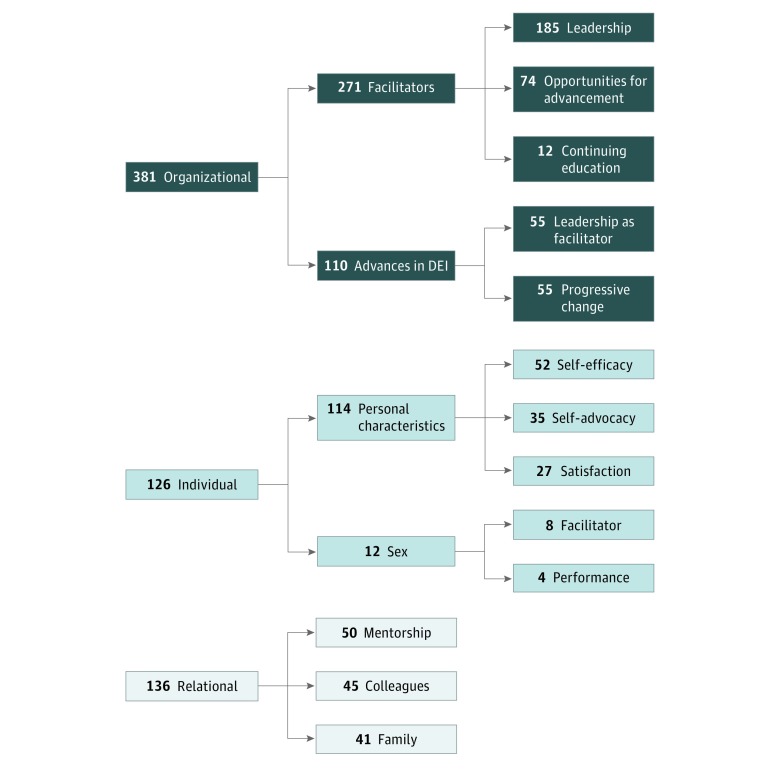
Diagram of Facilitator Codes (643 Citations): Thematic Narrative Analysis DEI indicates diversity, equity, and inclusion.

Major themes and subthemes are listed in the thematic analysis summary^17,18,19^ (Table 1). Major themes and predominant subthemes were categorized within the context of barriers and facilitators according to number and distribution of citations coded from participant interviews. Participant excerpts are included to illustrate these themes (Table 2). All participant names were anonymized and any potentially identifying information has been altered. Visual representations of barrier and facilitator themes and most-cited predominant subthemes are presented in flow diagrams. Figure 1 graphs the 1044 barrier citations and Figure 2 illustrates the 643 facilitator citations.

**Table 1.** Thematic Analysis Summary of Major Theme Definitions and Subtheme Categories of 26 Narrative Interviews

Theme	Definition	Subthemes
Institutional culture and institutional policies	Values within the organization that shape behavioral outputs and the structures, processes, and incentives in which it operates^17^	Defined barriers: negative culture, negative gendered environment, office politics, organizational barriers, clinical workloads, financial resources, institutional policies, faculty track, advancement limitations, attrition
		Defined facilitators: leadership development, advances in diversity and inclusion, leadership as facilitator, progressive change, opportunities for advancement, continuing education
Relational interactions	Interactions between agents and intersubjective ties between individuals^18^	Defined barriers: negative experiences with leadership, negative departmental relationships, challenges managing up, negative mentorship experiences, challenges managing down, unconscious bias, sex bias, negative experiences with colleagues
		Defined facilitators: positive mentoring, importance of collegial relationships, family support, positive leadership experiences
Individual characteristics	Dispositional, habitual, and motivational traits discernable to an individual that explain and predict behavior^19^	Defined barriers: confidence gap, naivete, personal limitations, unequal work-life balance, behaviors based on perceived expectations, questioning of sex bias, sex as hindrance
		Defined facilitators: self-efficacy, self-advocacy, hard-working, work-life prioritization, career satisfaction, professional growth, positive reputation, sex as opportunity

**Table 2.** Thematic Analysis Summary of Illustrative Quotes of 26 Narrative Interviews

Theme	Illustrative Quote
Institutional culture and institutional policies	Barrier: “There is less respect for those that do research and are not in the instructional track. Faculty in the clinical track are expected to do research, but I don’t have much protected time for that. The clinical work takes over and the limited time has been a barrier to moving forward with my research.” –Participant 143
	Facilitator: “There was an environment of not including women at the decision-making level, which appears to be changing. All the things that have started changing since I think are all good news. The fact that they’re recognizing they don’t have a lot of women residents. The fact that there are more women speakers at Grand Rounds. Those are all things that I certainly noticed here that I thought were different from where I came from.” –Participant 132
Relational interactions	Barrier: “I think many of the barriers are from our support. If my clinical support is asking me to do 10% or 15% more than they would ask the male colleagues to do, then that’s 10 to 15% of my time that I have to spend doing the clinical work that I don’t have for other things. I think that’s been largely neglected in many of the studies.” –Participant 146
	Facilitator: “My mentor supported me in everything I’ve done. They did everything possible to make sure that I was able to pursue what I needed to pursue to excel. My mentor was the one that if there were any problems or if I ever had any questions or concerns, they’d be the one I’d call.” –Participant 127
Individual characteristics	Barrier: “You wonder whether or not you’re being punished in other ways for standing up for yourself. That happened to me in terms of the negotiation. You don’t want to be perceived as greedy or trying to get more than you deserve. I don’t think I am being selfish, but I worry about the perception. I mean, especially if you have a valid concern to bring up. You almost have to think twice; you can’t always be the person kind of rocking the boat.” –Participant 144
	Facilitator: “My personal experience has been amazing. I always joke that the biggest problem with Michigan is that there are really no barriers to promotion, and you’re your own limitation. When you fail, it’s your fault. And there’s nothing quite as humbling as when you can’t blame a system or an organization or another person for your failures. It’s all on you. So, I feel like people here have gone out their way to give me every opportunity. For me, it’s been a really big growing experience.” –Participant 161

### Organizational Culture and Institutional Policies Affect Opportunities for Advancement

The most frequently cited inhibitors to advancement came from negative organizational culture and institutional policies that participants perceived impeded opportunity. We define organizational culture as values within the organization that shape behavioral outputs and institutional policies as the structures, processes, and incentives in which the institution operates.^17^ Culture and policies regarding negotiation, onboarding, promotion parameters and processes, independent funding, protected time, and clinical duties were important considerations for participants.

A salient experiential narrative within this theme is one of invisibility—being overlooked, left out, or unrecognized. The most highly cited barriers by our participants included the following:

- Participants felt tension between clinical and research responsibilities. Frustration was expressed as clinical work was perceived to be not highly valued in terms of advancement. Participants lamented that their clinical workload was often in conflict with their research time, regardless of expressed limits in contracts and grant documents for protected time. Participants frequently noted more women tend to be on the clinical track.
- Policies centered around faculty track were identified as problematic. Proper alignment for research, instructional, and clinical faculty tracks is critical for advancement and institutional success. Participants reported being slotted into tracks they felt were not optimal for workplace satisfaction or career goals; inconsistency of treatment for faculty on the clinical track compared with research track or tenure track; and the lack of flexibility to transfer, modify, or exit these tracks.
- Several participants cited salary inequities. Furthermore, the salaries of female surgeons were reported as being less than those of their male counterparts.

Encouragingly, many participants reported the perception of positive change over time in organizational culture and institutional policies to better support and facilitate advancement for women.

The facilitators to advancement were not as frequently cited in this category. However, these facilitators were viewed as being highly impactful on participant careers, particularly the following:

- The appointments of women to leadership positions. These appointments were seen as not only furthering equitable representation but also engendering much-desired and deserved visibility for female surgeons.^20,21^
- Leadership opportunities and leadership development, perceived as core facilitators for professional progress.
- The intentional focus on sex inclusivity being implemented through emerging diversity, equity, and inclusion efforts.

### Relational Interactions With Leadership, Mentors, Colleagues, and Staff Affect Attrition and Promotion

Relational barriers were highly cited. Relationality is defined as interactions between agents and intersubjective ties between individuals.^18^ Women in our study spoke of interactional bias at every level—leadership, residents, physician assistants, and nursing and administrative staff. Participants in our study frequently questioned whether sex bias was at the root of discrepancies for themselves and other women in regard to promotion, salary, administrative appointments, research funding, and collaborations.

The most highly cited relational barriers included the following:

- Relational barriers concerning negative relationships with department leadership were often reported. Participants found it particularly challenging to manage up. Participants stated that oppositional relationships with leadership, which felt personal, compromised their advancement.
- Participants highlighted challenging relationships with nursing staff, physician assistants, and other surgical support staff that were informed by sex, the management of which complicated their work.
- Lack of proper mentorship, specifically few female research mentors and unconstructive mentorships, was problematic.
- Negative sex-based interactions with male students and female peers were discussed.

Positive relationships at all levels were reported as requisite for female surgeons to garner success as academician, teacher, and clinician.^22^ Supportive partnerships at the supervisory and peer levels provide vital opportunities and valuable counseling. Constructive learner and staff relations are equally as important and contribute to a sense of personal success and well-being. The most frequently cited relational facilitators included the following:

- Mentor relationships were viewed as a powerful feature for advancement. Overall, participants were pleased with their mentorship opportunities and relationships.
- The strength of peer collaboration, encouragement, and guidance were reported as some of the most valuable facilitators for personal and professional satisfaction. Peer advocacy and sponsorships were often cited as factors that facilitate advancement.

Participant perceptions of their gendered relational experiences were disparate. Some reported experiencing an openly sexist environment where “Gender seemed to be an issue. It’s just the comments made by some males. But that’s just the way it is in surgery and I don’t think it is unique to Michigan.” (Participant 147).

Others noted seeing bias but not personally experiencing sex discrepancies. For example, “I think there is a fair amount of sexism. But I don’t think it’s deliberate. I think it’s unconscious bias. I never felt like I was picked on or treated differently or treated badly.” (Participant 151).

There were some participants who did not feel sex discrimination at all. One participant explained, “I don’t think that I’ve felt any gender-related bias here. On the contrary, I think I had the unique opportunity to observe a lot of really impressive women leaders who shaped me and allowed me to feel that I could do whatever it is that I wanted to do.” (Participant 160).

### Individual Characteristics Mediate the Perception of Professional and Personal Success

This theme addresses individual characteristics, which describe the dispositional, habitual, and motivational traits discernable to an individual that can help explain and predict behavior.^19^ It was stated repeatedly that surgery is a difficult culture. How participants perceived barriers and how they reacted to obstacles varied. Many of our participants reported that surgery was an inherently challenging field where surgeons must find internal fortitude to succeed. Therefore, participants often chose to subvert personal concerns and personality elements that might be perceived as incongruent with surgical culture expectations. Most frequently reported individual barriers included the following:

- Participants spoke of the requirement to “tough it out,” “not be a complainer,” and “keep their head up” when faced with career difficulties that were often perceived as personal.
- Many participants reported feelings of self-doubt, undervaluing their contributions.
- A common element in participant narratives is the perception of being “lucky.” Many participants attributed their appointments, advancements, and rewards to luck.
- Familial responsibilities and the difficulties of maintaining a satisfying work-life balance were cited. Challenges included pregnancy, maternity leave, and work-related conflict with partners.

One of the most common individual facilitators among participants was self-efficacy. Participant narratives that were centered in self-efficacy allowed those participants to create measures of personal resilience, purpose, and satisfaction when challenges presented themselves.^23^ The most frequently highlighted facilitators associated with individual characteristics included the following:

- Many participants identified the facility of finding or creating their own opportunities for growth and advancement.
- Individual responsibility to manage their environment and boost self-confidence to confront obstacles was frequently cited.
- Participants found satisfaction in the establishment of priorities and enforcing the boundaries surrounding them.

## Discussion

It’s not anything big that undermines women in academics. It’s many, many, many small inequities that build up over a career.

–Participant 150

In this quotation, a study participant succinctly captures the struggle for success of female surgeons. Our study found that a complex matrix of organizational and individual factors contribute to the sex inequities felt by women in academic surgery. Prohibitive institutional dogma and incompatible and unevenly applied policies represent the greatest impediment to advancement. However, relational dynamics and individual traits additionally contribute to promotional obstacles. Gendered experiences and the perception of sex bias are prominent features across this framework. The results of this investigation are unique, to our knowledge, as they have helped inform sex-based interventions that have shaped our departmental progress toward sex equity at Michigan Medicine. By modifying these specific factors to enhance the achievement and satisfaction of women in academic surgical medicine, we have actively integrated this research into practice and are seeing positive results in helping close the sex gap at Michigan (Table 3). Based on this evidence, it is our hope that institutions can create their own sex equity tool kit for surgery departments across the country.

**Table 3.** Examples of Early Outcomes of Developed Diversity Interventions at Michigan Medicine

Barrier	Intervention(s)	Outcomes (at 6 mo)	Representative Quote
Mentorship and sponsorship	Development of team-based mentoring (launch teams)	7 New launch team participants	“My launch team has constructed together diverse experts and leaders that embody the surgeon and academic that I want to become—including mentors from outside surgery and even from outside our institution.”
	Faculty Exchange Program	18 Faculty exchange visits across the country for young faculty to gain exposure	“As a midcareer surgeon it was really transformational (sabbatical)…I can’t imagine a better use of 2 months at this stage in my career to push me to a new level of understanding in advanced minimally invasive vascular surgery.”
	8-Week Sabbatical Program for midcareer faculty to acquire targeted professional development	6 Midcareer faculty participants in the sabbatical program	
Recruitment process	Formation of a diverse recruitment committee	Candidate pool diversification	“My experience with the recruitment committee solidified my decision to join the faculty. I felt welcome and included, and that people in the room cared about me as a person.”
	Formalized recruitment process mandating all positions to interview ≥1 woman and underrepresented minority	Increased faculty diversification (eg, race/ethnicity, sex, academic and clinical interests)	
Implicit bias	Department-wide implicit bias training	Entire department completed implicit bias training	“There is an emerging body of evidence that shows that when we are dealing with complex problems that having diversity in all of its forms leads to optimal solutions.”
	Grand Rounds diversity series	Increased recognition of diversity as a competitive advantage	“I think we have all come to learn and creatively think about things in a diverse environment that we physically and cognitively were not capable of doing in a less diverse environment.”

While institutions may not intend for their cultures or policies to inhibit the career development of women, we have found that time-honored traditions and unexamined biases strongly affected the experiences of female surgeons. Institutions must proactively identify policies that may be prohibiting promotion and could be making advancement particularly tricky for female surgeons.^24,25^ Proper organizational guidance, career support, and careful professional navigation could make advancement for women in surgery much more beneficial for the institution and the individual.^26,27^ The development of educational programs that augment leadership skillsets may provide women with the necessary skills to navigate institutional challenges and further equitable sex representation at the leadership level.^20,21^ At Michigan Medicine, more women are taking part in initiatives like the Leadership Development Program, a yearlong program that prepares faculty for institutional and professional society leadership, team building, and business acumen.

Relational interactions with leadership, mentors, colleagues, and surgical support and administrative staff can represent challenges to success. Experiences of sex bias remain a consequence of the perceived masculine domain of surgery.^18,28,29,30^ Our participants’ narratives communicate what often goes unsaid. Biased interactions are felt deeply; they erode trust, confidence, and effectiveness.^5^ A strong commitment to diversity, equity, and inclusion efforts that provide systematic training in diversity awareness and unconscious bias training can help promote a culture that recognizes the value of sex, racial, and cognitive diversity. We encourage departments to actively address unconscious bias and help educate faculty on strategies that can encourage diversity. The Michigan Medicine Department of Surgery promotes Strategies and Tactics for Recruiting to Improve Diversity and Excellence workshops. These workshops enhance awareness of unconscious bias and develop standardized processes and metrics to promote recruitment, aid individuals to challenge bias, and overcome blind spots.^31^ The workshops provide concrete resources to challenge bias on multiple fronts—including guidance on writing fair letters of recommendation, creating climates that strengthen retention for women and minorities, and providing career advice for junior and senior faculty.

Individual attributes of perseverance, drive, and strong work ethic are often identified as critical success factors for female surgeons.^32^ The need to add value, self-advocate, control your environment, know how to make things work, and self-direct your career path were characteristics recognized as providing advantages that help guard against negative experiences and career roadblocks.^28^ The implementation of interventions to boost confidence, eradicate the imposter syndrome, celebrate success, and deal with negative thoughts through formalized individual and group coaching should be a part of every department’s sex equity tool kit.^31,33^ Taking this lead, the MWSC works to advance female surgeons with an intentional emphasis on creating a supportive culture for female surgeons. The MWSC develops, disseminates, and implements strategies that promote the academic advancement of female surgeons to achieve diversity, equity, and inclusion across surgical disciplines. A key component of their efforts is an annual leadership development conference that fosters community, inclusivity, and growth. Women-centered faculty collaboratives, like MWSC, can foster cultures that actively support female surgeons as they work to attain career goals and pursue formidable positions with greater confidence.

### Limitations

There are limitations to this study. This analysis is based on narratives of 26 female faculty from a single academic surgery department, thus limiting the generalizability of these findings. Individuals self-identified to participate in this study; therefore, the possibility of self-selection bias exists. Data collected were limited to self-reported information focusing on academic advancement and gendered experiences. The coding and analysis of this data were operationalized within that context. This study was intentionally designed with the aim to gather the stories of female surgeons with the express goal of giving voice to the experiences and perspectives of female surgeons to identify issues and concerns specific to this group. Supplemental themes and perspectives derived from the content-rich participant narratives are worth further exploration. Future work on this subject aims to include the perceptions and experiences of surgeons across sexes and subspecialties to relate barriers and facilitators to academic advancement. We hope to extend the study to explore the identification of barriers and facilitators to achievement of male surgeons and, further still, to stratify the experiences of underrepresented minorities.

## Conclusions

A complex array of factors have contributed to the sex inequities felt by women in academic surgery at Michigan Medicine. Organizational culture and institutional policies are powerful forces in both the inhibition and the advancement of female surgeons. Relational interactions are felt deeply and any impediments require continual assessment and change. Individual characteristics grounded in self-efficacy provide support on a personal level and can be improved with leadership development and coaching. In addition, surgical departments must be reflective and willing to see institutionally ingrained sex bias. Women perceive that opportunities for advancement, collaboration, networking, and support are missed because of sex. Our work has practical implications, as it will inform and shape our departmental progress toward sex equity. We hope this research may help build theory on barriers and facilitators to academic advancement for female surgeons and help develop evidence-based interventions to close the sex gap in surgery departments across the country.

## References

[1] Kohona HEAD, Dolling DL, Foster E Empowering women and gender equality: minority and discrimination. https://www.lightmillennium.org/2011_27th/bircan_unver_marginilized_minoritized_final.pdf. Accessed October 26, 2011.

[2] Page SE Making the difference: applying a logic of diversity. Acad Manage Perspect. 2007;21(4):-. doi:10.5465/amp.2007.27895335

[3] Greenberg CC Association for Academic Surgery presidential address: sticky floors and glass ceilings. J Surg Res. 2017;219:ix-xviii. doi:10.1016/j.jss.2017.09.006 29078918

[4] Zhuge Y, Kaufman J, Simeone DM, Chen H, Velazquez OC Is there still a glass ceiling for women in academic surgery? Ann Surg. 2011;253(4):637-643. doi:10.1097/SLA.0b013e3182111120 21475000

[5] Bickel J Women’s career development: what does this have to do with men? Ann Surg. 2011;253(4):644-646. 21475001 10.1097/SLA.0b013e318211a8fe

[6] Andrews NC Climbing through medicine’s glass ceiling. N Engl J Med. 2007;357(19):1887-1889. doi:10.1056/NEJMp078198 17989380

[7] Jagsi R, DeCastro R, Griffith KA, Similarities and differences in the career trajectories of male and female career development award recipients. Acad Med. 2011;86(11):1415-1421. doi:10.1097/ACM.0b013e3182305aa6 21952061

[8] Waljee JF, Chang KW, Kim HM, Gender disparities in academic practice. Plast Reconstr Surg. 2015;136(3):380e-387e. doi:10.1097/PRS.0000000000001530 26313843 PMC4785879

[9] Cassell J The Woman in the Surgeon’s Body. Cambridge, MA: Harvard University Press; 1998.

[10] Butler J Performative acts and gender constitution: an essay in phenomenology and feminist theory. Theatre J. 1988;40(4):519-531. doi:10.2307/3207893

[11] Hawick L, Cleland J, Kitto S ‘I feel like I sleep here’: how space and place influence medical student experiences. Med Educ. 2018;52(10):1016-1027. doi:10.1111/medu.13614 29932224

[12] Whiteman G, Phillips N The role of narrative fiction and semi-fiction in organizational studies In: Barry D, Hansen H, eds. New Approaches in Management and Organization. London, UK: Sage; 2008:288-299.

[13] Creswell JW, Miller DL Determining validity in qualitative inquiry. Theory Pract. 2000;39(3):124-130. doi:10.1207/s15430421tip3903_2

[14] Denardo AM. Using NVivo to analyze qualitative data. http://citeseerx.ist.psu.edu/viewdoc/summary?doi=10.1.1.83.5090. Accessed March 2018.

[15] Watt D On becoming a qualitative researcher: the value of reflexivity. Qual Rep. 2007;12(1):82-101. http://www.nova.edu/ssss/QR/QR12-1/watt.pdf. Accessed April 2018.

[16] Braun V, Clarke V Using thematic analysis in psychology. Qual Res Psychol. 2006;3(2):77-101. doi:10.1191/1478088706qp063oa

[17] Denison DR Corporate Culture and Organizational Effectiveness. Hoboken, NJ: John Wiley & Sons; 1990.

[18] Bourdieu P, Passeron J-C Reproduction in Education, Society and Culture. 2nd ed. Thousand Oaks, CA: Sage Publications; 1990 Theory, Culture & Society; vol 4.

[19] Anderson N, Ones DS, Sinangil HK, Viswesvaran C, eds. Personnel Psychology. Thousand Oaks, CA: Sage Publications; 2001 Handbook of Industrial, Work & Organizational Psychology; vol 1.

[20] Fernández ML, Castro YR, Otero MC, Foltz ML, Lorenzo MG Sexism, vocational goals, and motivation as predictors of men’s and women’s career choice. Sex Roles. 2006;55(3-4):267-272. doi:10.1007/s11199-006-9079-y

[21] Rudman LA Sources of implicit attitudes. Curr Dir Psychol Sci. 2004;13(2):79-82. doi:10.1111/j.0963-7214.2004.00279.x

[22] Shen H Inequality quantified: mind the gender gap. Nature. 2013;495(7439):22-24. doi:10.1038/495022a 23467149

[23] Bandura A Perceived self-efficacy in cognitive development and functioning. Educ Psychol. 1993;28(2):117-148. doi:10.1207/s15326985ep2802_3

[24] Strong EA, De Castro R, Sambuco D, Work-life balance in academic medicine: narratives of physician-researchers and their mentors. J Gen Intern Med. 2013;28(12):1596-1603. doi:10.1007/s11606-013-2521-2 23765289 PMC3832709

[25] De Simone S, Scano C Discourses of sameness, unbalance and influence: dominant gender order in medicine. J Gend Stud. 2017;27(8):914-927. doi:10.1080/09589236.2017.1357541

[26] Colletti LM, Mulholland MW, Sonnad SS Perceived obstacles to career success for women in academic surgery. Arch Surg. 2000;135(8):972-977. doi:10.1001/archsurg.135.8.972 10922261

[27] Sonnad SS, Colletti LM Issues in the recruitment and success of women in academic surgery. Surgery. 2002;132(2):415-419. doi:10.1067/msy.2002.127694 12219043

[28] Hill E, Solomon Y, Dornan T, Stalmeijer R ‘You become a man in a man’s world’: is there discursive space for women in surgery? Med Educ. 2015;49(12):1207-1218. doi:10.1111/medu.12818 26611186

[29] Bourdieu P, Wacquant LJ An Invitation to Reflexive Sociology. Chicago, IL: University of Chicago Press; 1992.

[30] Lizardo O The cognitive origins of Bourdieu’s habitus. J Theory Soc Behav. 2004;34(4):375-401. doi:10.1111/j.1468-5914.2004.00255.x

[31] Dossett LA, Mulholland MW, Newman EA; Michigan Promise Working Group for Faculty Life Research Building high-performing teams in academic surgery: the opportunities and challenges of inclusive recruitment strategies. Acad Med. 2019. doi:10.1097/ACM.0000000000002647 30730376

[32] Kass RB, Souba WW, Thorndyke LE Challenges confronting female surgical leaders: overcoming the barriers. J Surg Res. 2006;132(2):179-187. doi:10.1016/j.jss.2006.02.009 16564542

[33] Newman EA, Waljee J, Dimick JB, Mulholland MW Eliminating institutional barriers to career advancement for diverse faculty in academic surgery. Ann Surg. 2019;270(1):23-25. doi:10.1097/SLA.0000000000003273 30946081

